# Identification of the body fluid donors from mixture stains using bulk transcriptomes data

**DOI:** 10.1093/bib/bbaf668

**Published:** 2025-12-12

**Authors:** Huan Yu, Jiayan Li, Jiaxin Ji, Jiaqi Wang, Zidong Liu, Hailing Yang, Xiaoxiao Wang, Jinding Liu, Zeqin Li, Gengqian Zhang

**Affiliations:** School of Forensic Medicine, Shanxi Medical University, No. 55 Wenhua Street, Yuci District, Jinzhong, Shanxi 030619, China; School of Forensic Medicine, Shanxi Medical University, No. 55 Wenhua Street, Yuci District, Jinzhong, Shanxi 030619, China; School of Forensic Medicine, Shanxi Medical University, No. 55 Wenhua Street, Yuci District, Jinzhong, Shanxi 030619, China; School of Forensic Medicine, Shanxi Medical University, No. 55 Wenhua Street, Yuci District, Jinzhong, Shanxi 030619, China; School of Forensic Medicine, Shanxi Medical University, No. 55 Wenhua Street, Yuci District, Jinzhong, Shanxi 030619, China; School of Forensic Medicine, Shanxi Medical University, No. 55 Wenhua Street, Yuci District, Jinzhong, Shanxi 030619, China; School of Forensic Medicine, Shanxi Medical University, No. 55 Wenhua Street, Yuci District, Jinzhong, Shanxi 030619, China; School of Forensic Medicine, Shanxi Medical University, No. 55 Wenhua Street, Yuci District, Jinzhong, Shanxi 030619, China; School of Forensic Medicine, Shanxi Medical University, No. 55 Wenhua Street, Yuci District, Jinzhong, Shanxi 030619, China; School of Forensic Medicine, Shanxi Medical University, No. 55 Wenhua Street, Yuci District, Jinzhong, Shanxi 030619, China

**Keywords:** forensic genetics, body fluid mixture, mixture deconvolution, transcriptome, genetic variation

## Abstract

The deconvolution of the mixture contributors is essential but always presents challenges in crime scene investigation, especially when encountering mixtures with multiple contributors. In recent years, RNA-based genotyping has shown great advantages in assigning a body fluid to a specific mixture contributor, whereas transcriptome sequencing has been seldom applied for mixture deconvolution. In this study, we investigated the feasibility of bulk transcriptomes data for deconvoluting biological mixtures, and described a novel approach, utilizing bulk transcriptomes data of multi-body-fluid mixtures to identify body fluid donors. Computational deconvolution methods were introduced here to infer body fluid proportions from bulk transcriptomes data, and SNPs corresponding to a specific body fluid donor could be separated from differentially expressed genes with the knowledge of body fluid composition. The described method was tested on both *in-silico* and real mixtures. After a quantitative evaluation, robust regression-based deconvolution methods with unnormalized expression profiles were finally applied to body fluid deconvolution tasks, and body fluid compositions of mixtures could be determined with high accuracy according to the deconvolution results. Finally, the corresponding SNPs were successfully extracted from both *in-silico* and real mixtures, producing high likelihood ratios and strong evidentiary weight for individually identifying body fluid donors. Altogether, our proof-of-concept study established the utility of bulk transcriptomes data to identify mixture contributors, providing new avenues for mixture deconvolution.

## Introduction

Biological mixtures containing two or more individual body fluids are commonly encountered in crime scenes. Deconvoluting these biological mixtures has long been considered a main challenge in forensic casework and various biological research fields [1]. As the gold standard for human identification, Short Tandem Repeat (STR) polymorphisms are routinely used for analyzing biological evidence [2]. However, their application for mixture analysis proved to be limited, due to the great complexity of deconvoluting mixed STR profiles even when combined with probabilistic genotyping methods [3–5]. The methods that separate mixture components and generate individual profiles, by contrast, seem more promising.

Several physical separation methods prior to DNA profiling have been proposed by forensic scientists, including differential lysis [6], which was used for separating sperm and vaginal epithelial cells, and methods for separating single cells, such as DEPArray™ [7] or laser capture microdissection [8]. However, differential lysis is restricted by the incomplete separation of male and female components [9, 10], and single-cell separation technologies still present challenges for the sensitivity of current analytical approaches [11, 12]. Another separation strategy is to target unique genomic regions of a specific contributor [1]. This approach allows the DNA of different contributors to be selectively amplified, thereby enabling the generation of individual genetic profiles. Y-chromosome markers [13] and a category of compound markers, such as pairing deletion/insertion polymorphisms (DIP) with STR or SNP [14, 15], were both developed based on this strategy to target the unique alleles of a specific donor. These markers take effect only when the donor possesses a unique allele, which limits their application for multi-donor mixtures.

In recent years, transcriptome analysis has experienced tremendous growth and brought substantial progress to forensic genetics. Although RNA is generally considered thermodynamically unstable, it can still be recovered from casework samples with sufficient quality and quantity for forensic analysis [16]. Forensic RNA analysis has therefore emerged as a useful tool to identify body fluids [17–19], and genotyping of SNPs present in body fluid-specific RNAs makes it possible to directly associate a body fluid with a specific individual [20–22]. These all enable the RNA-SNP marker as a novel separation strategy to acquire individual profiles from biological mixtures.

Several studies have successfully generated whole transcriptome data from forensic samples [23–25]. Transcriptome sequencing allows for the simultaneous detection of tissue-specific transcript levels and sequence variations within RNA molecules. It seems that transcriptome data also shows potential to provide a direct link between the suspect and the source of body fluid. Nonetheless, to the best of our knowledge, transcriptome sequencing has not yet been applied to simultaneously identify body fluids and their contributors in a mixture.

**Figure 1:**
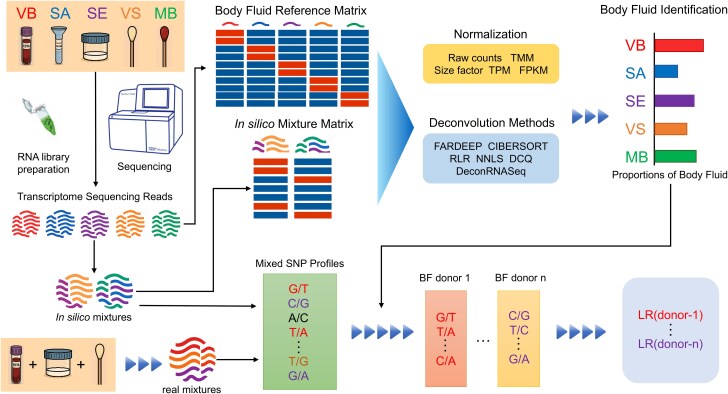
Schematic representation of the design of this study.

Here we performed whole transcriptome sequencing on forensic body fluid samples, including venous blood (VB), semen (SE), saliva (SA), vaginal secretion (VS), and menstrual blood (MB). Reads of body fluid transcriptomes were randomly selected, and the sampling reads from different body fluids were combined to generate *in-silico* transcriptomes of mixtures. Based on the current datasets, we investigated the availability of bulk transcriptome data for deconvoluting multi-body-fluid mixtures, and described a deconvolution approach based on a series of bioinformatic methods. The method was assessed on both artificial transcriptomes and mRNA-seq data of real mixtures. Using this approach, we successfully inferred the number and origin of contributed body fluids in mixed samples and assigned each inferred body fluid to a specific individual (Fig. 1).

## Materials and methods

### Sample collection and RNA preparation

Five forensically relevant body fluids were collected from healthy volunteers. Specifically, venous blood samples were drawn from five unrelated volunteers by cephalic venipuncture and stored in vacutainer tubes. Freshly ejaculated semen samples (n = 5) were donated in screw-cap containers. Liquid saliva samples were donated in sterile tubes from nine unrelated individuals, after they had refrained from drinking or eating for at least 1 hour. All liquid samples were placed into a −80°C freezer immediately after the samples were transported to our laboratory. Vaginal secretion and menstrual blood samples (the second day of the period) were collected by using sterile cotton swabs from the vagina of five healthy unrelated women, respectively. All swab samples were dried in the dark for at least 12 hours before being stored at −80°C. This study was approved by the Ethics Committee of Shanxi Medical University, China (2021GLL052), with written informed consents provided by all volunteers.

At least 1 ml of venous blood, semen and saliva or two swabs of vaginal secretion and menstrual blood were used for RNA extraction. Total RNA was isolated with RNAiso plus (Takara Biomedical Technology, China) according to the manufacturer’s instructions. The RNA dissolved in RNase-free water was quantified by using Nanodrop 2000 (Thermo Fisher Scientific, Massachusetts, USA), and the purity of RNA samples was determined with the ratio of A260 and A280.

### Library construction

The Ribo-off rRNA Depletion Kit (Human/Mouse/Rat) (Vazyme, China) was used to selectively deplete human ribosomal RNA (rRNA). Considering the high occupation of microbiome RNA in total RNA extracted from saliva and vaginal secretion, Ribo-off rRNA Depletion Kit (Bacteria) (Vazyme, China) was additionally applied for the depletion of microbial rRNA to improve the acquisition of human-derived reads. The RNA libraries were subsequently constructed by using U-RNAseq Library Prep Kit on 20 individual body fluid samples, including venous blood, semen, vaginal secretion and menstrual blood, and three saliva samples each pooled by saliva from three individuals. The quantities and insert size of RNA libraries were assessed by using Qubit 3.0 fluorometer (Invitrogen, USA) and Agilent 2100 Bioanalyzer (Agilent Technologies, USA), respectively, and qualified libraries were sequenced on a NovaSeq6000 sequencer (Illumina, USA) with the NovaSeq 6000 SP Reagent Kit using 2 × 150 bp run configuration.

### Generation of *in-silico* mixtures with multiple body fluid components

Transcriptome data of *in-silico* mixtures were generated for the evaluation of mixture deconvolution pipeline. Seqtk v1.4 was used for randomly sampling a specific number of reads from body fluid transcriptome data. For two-person mixtures (n = 180), each artificial mixture was created by randomly selecting two body fluid types to be present, followed by choosing a proportion of reads assigned to each body fluid (enforcing a sum-to-one constraint) among all possible proportions between 0.1 and 0.9, in increasing intervals of 0.1. For multi-person mixtures (n = 30), *in-silico* mixtures were generated with the same proportion of reads randomly sampled from three or four body fluid types. All the body fluid samples used for generating *in-silico* mixtures, as well as their proportion of reads within mixtures, were summarized in Supplementary Table S1, and each mixture was generated with a total of 56 million reads.

### RNA bioinformatic analysis

Trim-galore v0.6.10 was used to filter remnant adapter sequences, short reads (length < 36 bp) or low-quality reads (Q < 25) and save clean reads into FASTQ format. Clean fastq-files were then aligned to the human genome (GRCh38) using the STAR aligner v2.7.11a [26]. Obtained bam-files were used for the discovery of SNPs and Indels based on the Best Practice Workflow provided by GATK, and all the procedures were performed using the commands of GATK v4.6.1.0 according to the default parameters, including marking duplicated reads, reformatting alignments spanning introns, base quality score recalibration, and short variant genotype calling [27]. Gene expression level was calculated as read counts, fragments per kilobase million (FPKM), and transcripts per million (TPM) by using RSEM v1.3.3 [28]. Differential expression analysis was performed using DESeq2 v1.46.0 in R [29].

### Generation of reference matrices for body fluid deconvolution

The computational methods to infer cell type proportions from bulk RNA-seq data were introduced here to deconvolute body fluid proportions in mixture transcriptomes. We first used body fluid transcriptome datasets to constitute input matrices of reference signatures, in which average expression level across samples from each body fluid type was computed for each gene. The reference genes used to distinguish body fluids could be obtained by differential expression analysis. Differentially expressed genes (DEGs) between pairwise body fluid types were identified with an absolute fold change ≥2 and q-value <0.05. For each body fluid type, significant DEGs were ordered by decreasing absolute fold change compared to other body fluid types, and top DE genes from each pairwise type were combined into a reference matrix. We iterated the number of selected genes from the top 30% of DEGs to 1% to explore the matrices with maximum performance. Furthermore, a total of four normalization methods, including FPKM, TPM, size factor normalization (provided by DEseq2 package [29]) and TMM normalization (provided by edgeR package [30]), were implemented on reference matrices and expression matrices of *in-silico* mixtures, and the deconvolution performance across four normalization strategies and original raw counts was measured to investigate the impact of data normalization.

### Measures of various deconvolution methods

Six mathematical methods used for deconvolution of bulk transcriptome data have been measured, including non-negative least squares (NNLS), robust linear regression (RLR), FARDEEP [31] based on robust regression, Digital Cell Quantifier (DCQ) [32] based on penalized regression, a quadratic programming DeconRNASeq [33], and CIBERSORT [34] based on support-vector regression. The concordance between known proportions of body fluids (expected proportions) and estimated proportions (observed proportions) was measured by root-mean-square error (RMSE) and Pearson correlation coefficient (R). These two values could clearly exhibit the estimation bias and linear fit of various deconvolution methodologies combined with different normalization strategies.

### SNP selection for identification of body fluid donors

The SNP loci called from transcriptomes of body fluid samples or *in-silico* mixtures were carefully selected before being applied to individual identification. Firstly, only bi-allelic autosomal SNPs fulfilling the genotype quality thresholds of read depth (DP) > 60 and genotype quality (GQ) > 40 were retained from the SNP list called by GATK. SNPs located in reported imprint genes or SNPs with A-to-I RNA editing events in REDIportal [35] were both excluded. Secondly, SNPs had to be covered by the 1000 Genomes Project Phase 3 datasets [36], and a minor allele frequency (MAF) threshold of >0.1 in the East Asian population was set to ensure the discrimination power of candidate SNP loci.

For the original datasets from individual samples, candidate SNPs were additionally assessed for statistical independence by using PLINK v1.90. A pruning step was performed to select SNPs with few effects of linkage disequilibrium (LD) using a 1000 kb distance between included SNPs and a correlation coefficient (r^2^) threshold of 0.2. The genotype distribution of retaining SNPs should also show no deviation from Hardy–Weinberg equilibrium (*P* > .05) in the East Asian population. The final set of SNPs was used for the calculation of total random match probability (RMP) according to the formula previously reported [12], in which genotype frequencies were accessed from 1000 Genomes Project Phase 3 datasets.

For the datasets of mixtures, only SNPs located in relatively specific genes were retained from the candidate SNP list. Specifically, for two-person mixtures, DEGs between two contributed body fluids would be characterized as relatively specific genes, and SNPs located in these genes would be directly selected. The composition of mixtures has already been determined through the deconvolution methods mentioned above. However, for mixtures containing three or four individuals, only the genes overexpressed in a body fluid component compared to any of the other components were characterized as relatively specific genes. The SNPs located in the same gene were considered as linked SNPs and recorded as phenotypes according to the approaches mentioned in our previous study [20]. Phenotype information of these SNPs was also accessed from 1000 Genomes Project Phase 3 datasets and applied for the calculation of the likelihood ratio (LR), which could be directly calculated from the RMP as


$$ LR=\frac{1}{RMP} $$

Where we assumed that the used genotypes were derived from a single body fluid donor and perfectly matched with reference individual samples, and there was a scenario with no allelic dropout/drop-in or other errors.

### mRNA-seq of real multi-body-fluid mixtures

Two laboratory-made mixtures were generated by mixing total RNA of venous blood, vaginal secretion and semen samples. The VB-VS mixture is mixed with equal amounts of total RNA of venous blood and vaginal secretion, and the VB-VS-SE mixture is mixed by total RNA of venous blood, vaginal secretion and semen with a ratio of 1:2:1. The mRNA was purified from these real mixture samples, and the libraries were prepared by using Fast RNA-seq Lib Prep Kit (ABclonal, China). After the quality control, quantified libraries were pooled and sequenced on a NovaSeq6000 sequencer (Illumina, USA). The sequencing results were analyzed using the method mentioned above.

### Analysis of bi-parental biogeographic ancestry

We first applied the above SNP deconvolution strategy to obtain candidate SNPs from transcriptome datasets of mixtures. A total of 2504 individuals from five continental populations were accessed from 1000 Genomes Project Phase 3 and applied as reference populations. The MAF threshold was discarded, and potential ancestry informative SNPs (AISNPs) were selected based on the criteria of allele frequency differences >0.3 in any pair of continental populations and adjusted for linkage disequilibrium (LD) using the method mentioned above. STRUCTURE v2.3.4 was used to evaluate individual ancestral proportions based on the selected AISNPs with 10,000 burn-ins and 10,000 Markov Chain Monte Carlo steps under the admixture model [37]. K was set from 2 to 6, and the optimal K value was evaluated using the Structure Harvester Program [38].

## Results

### Overview of transcriptome sequencing results

The quality control of total RNA and its sequencing results is summarized in Supplementary Table S2. The concentration of total RNA from all body fluid samples ranged from 29.94 ng/μl to 858.86 ng/μl, with A260/A280 ranging from 1.25 to 1.85. Vaginal secretion samples showed significantly higher RNA acquisitions compared to other types of body fluid samples. An average of 78.79 M (million) raw reads was obtained from 23 samples. The average Q30 value across all samples was 93.40% (88.90% ~ 95.65%), suggesting good sequencing quality. After data filtering, clean reads were aligned to the human genome, and highly varied mapping rates were detected from different body fluids. High and stable uniquely mapping rates could be identified from venous blood samples, while other body fluids showed obvious fluctuation of mapping rates. MB_10, SE_7 and VS_6 showed significantly lower mapping rates compared with samples from the same group, which were consequently excluded from differential expression analysis.

### The feasibility of individual identification using transcriptome data

**Figure 2:**
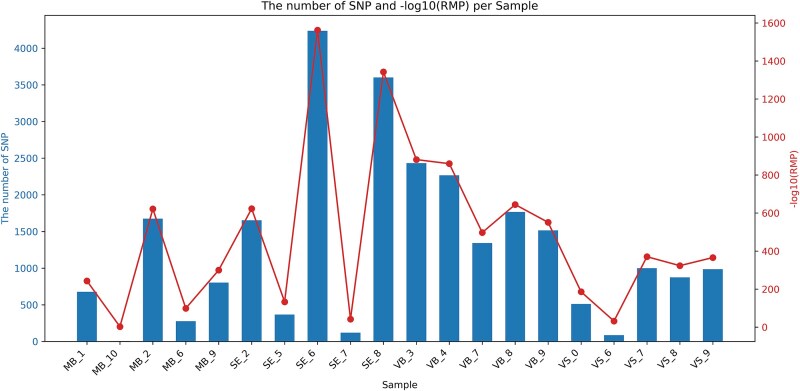
The performance from individual identification using individual transcriptomes of forensic body fluids, which is illustrated by random matching probability (RMP).

We initially investigated the potential of RNA-seq data for human identification and the strength of evidence it could provide. Transcriptome datasets of 20 individual samples were used, and the RMP value was applied as a statistical parameter, indicating the probability of the sample identically matching with an unrelated individual. For all datasets from 20 individual samples, a DP threshold of >60 was set to ensure high-confidence of SNP genotypes, and LD effects have been mitigated by only retaining the higher polymorphic SNP from each pair of SNPs in LD (r^2^ > 0.2). Apart from three samples with extremely low mapping rates (MB_10, SE_7 and VS_6), the number of polymorphic SNPs from these RNA-seq datasets ranged from 276 to 4236, and calculated RMP values ranged from 2.45E-99 to 7.24E-1563 (Fig. 2). However, even the SE_7 and VS_6 shown with extremely low mapping rates of 2.94% and 3.22%, they still obtained RMP values of 2.29E-42 and 2.69E-32, respectively, which were expected to yield similar high LRs and provide enough evidentiary weight when matching identity SNPs against a reference sample. These results successfully demonstrated the feasibility of using RNA-seq data, including the low-quality data, for the determination of human identity.

### Method for deconvoluting multi-body-fluid mixtures using bulk transcriptome data

**Figure 3:**
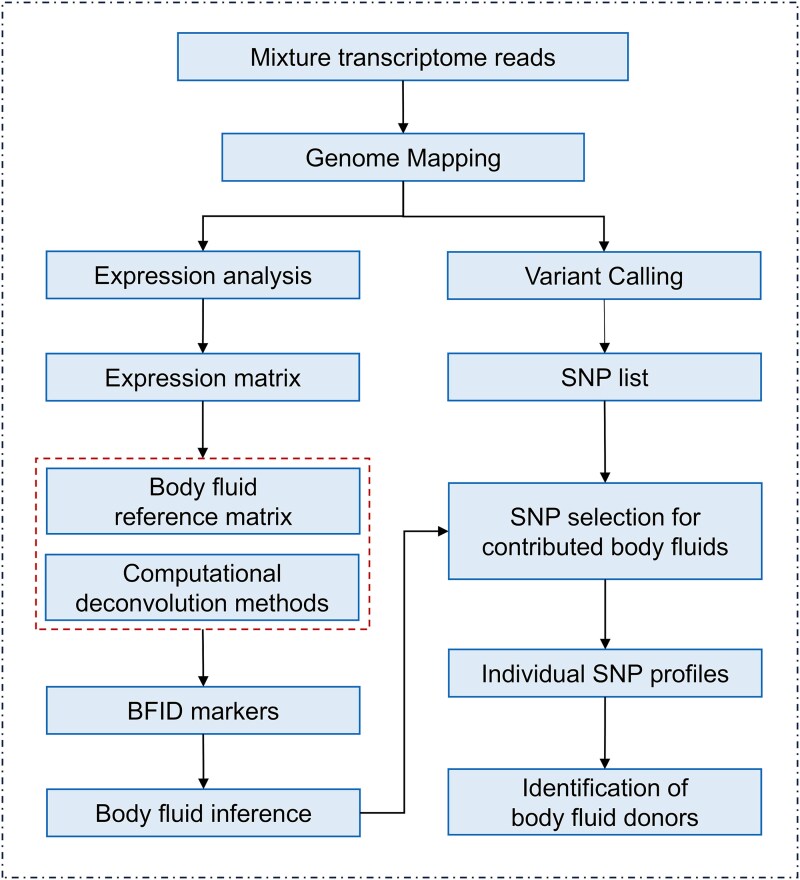
The workflow for deconvoluting multi-body-fluid mixtures with bulk transcriptomes data in two steps (body fluid type deconvolution followed by separation of SNP profiles corresponding to body fluid donors).

Next, we probed into the possibility of using bulk transcriptome data to genetically separate contributors from a mixture. A novel approach was therefore described, which was shown in Fig. 3. The mixture transcriptome data were first generally aligned to the human genome to obtain expression matrices and SNP calling lists. However, these SNPs called from mixture transcriptome could not be directly applied for individual identification, since most of them are observed with mixed genotypes from several persons. On the other hand, it is also of great importance to determine the mixture composition, as it provides the evidence for source-level propositions. The computational deconvolution methods were introduced here to infer body fluid proportions and determine body fluid composition in a mixture. The expression level of body fluid identification (BFID) markers in mixture expression profiles was also applied to assist in the inference. With the knowledge of body fluid compositions, a selection strategy was therefore developed, and SNPs located in relatively specific genes would be identified as corresponding SNPs to this body fluid donor. Consequently, individual SNP profiles were obtained from mixture transcriptome data, achieving the identification of body fluid contributor.

### Combined impact of factors on inferring body fluid proportions

**Figure 4:**
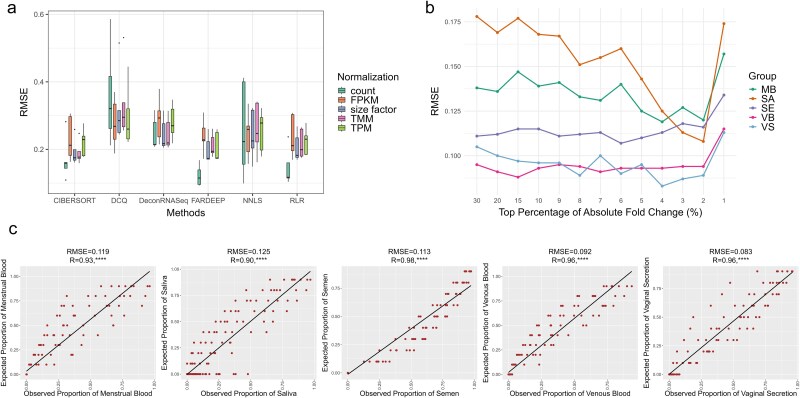
The body fluid deconvolution results with computational deconvolution methods. (a) Combined impact of deconvolution methodologies and normalization strategies on the deconvolution results. (b) Impact of marker selection on the deconvolution results. (c) The deconvolution results of the read fraction of each body fluid type using the deconvolution strategy with maximum performance. The deconvolution performance is assessed by root-mean square error (RMSE) and Pearson correlation coefficient (R) between known proportions of each body fluid (expected proportions) and predicted proportions from computational deconvolution methods (observed proportions).

Many mathematical methodologies have been developed to infer cell type proportions. A series of factors have also been reported to affect the deconvolution task, including data transformation, normalization, marker selection, and composition of transcriptomes [39, 40]. In this study, these computational methods were introduced for deconvoluting body fluid read proportions, and the combined impact of some factors was also assessed to investigate a deconvolution strategy with maximum performance. We first focused on the overall performance of six deconvolution methods across five normalization strategies. The number of markers selected for reference matrices was primarily set as the top 10% of DEGs ordered by decreasing absolute fold changes from each pairwise type. Two-person artificial mixtures generated with various body fluid proportions were used as input datasets. The results were shown in Fig. 4a. CIBERSORT, FARDEEP and RLR consistently showed better performance (lower RMSE values) than other three methods, while DCQ led to the worst results, with RMSE values for MB consistently higher than 0.4. The choice of normalization strategy did not have a substantial influence on the deconvolution results of most methods, except for FARDEEP and RLR. These two robust regression methods combined with unnormalized datasets outperformed other combinations a lot, achieving RMSE lower than 0.12. Downstream analyses were therefore performed using FARDEEP on data in an original unnormalized scale.

Next, we investigated the impact of marker selection on the deconvolution task. The number of selected genes was iterated from the top 30% to 1%. As the number of markers decreased, minor impact was shown in deconvolution performance until the marker was restricted to the top 1% of DEGs (Fig. 4b). Since a reference matrix with more markers required higher memories and took more times to complete the analysis, the top 4% of DEGs from each pairwise type was finally selected for generating body fluid reference matrix. The reference signatures, as well as their average expression levels in each body fluid type, were summarized in Supplementary Table S3.

### Deconvolution results with the strategy of maximum performance

After ascertaining the choice of methodologies, normalization and marker selection strategy, we benchmarked this deconvolution pipeline on all artificial mixtures, and the estimated proportions for five body fluids were summarized in Supplementary Table S4. We first compared the estimated proportions to known fractions of five body fluids on two-contributor mixtures with varying body fluid proportions (Fig. 4c). The inferred proportions of all body fluids were significantly correlated with the given proportions (R > 0.9, *P* < .0001), with an RMSE value ranging from 0.083 to 0.125. These results demonstrated a high level of consistency between the known and predicted proportions for each body fluid.

Then we interrogated the accuracy of this strategy for determining the presence or absence of a body fluid. All two- or multi-body-fluid mixtures were used for this evaluation, and an estimated proportion of >5% was considered as the presence of a specific body fluid. High overall accuracy was observed in four body fluid types, except for the determination of saliva, which exhibited a poor performance with a sensitivity of 86.96% and a specificity of 79.66% (Table 1). We observed that the false positive determination of saliva was mostly derived from mixtures generated from vaginal secretion or menstrual blood (Supplementary Table S4). However, even though estimated with high saliva fractions, these samples showed no expression (read counts <10) of saliva-specific genes, such as STATH, whose expression could be easily observed in saliva-contributed mixtures. Considering that body fluid identification results hold key roles in the following SNP selection strategy, we therefore recommend determining body fluid composition by combining deconvolution results with the expression levels of highly specific BFID markers to assist in the inference and improve the accuracy. The well-validated BFID markers, which also showed superior specificity in our datasets, are summarized in Table 2.

**Table 1:** The accuracy of the deconvolution strategy for determining body fluid composition.

Body fluid	TP	TN	FP	FN	Sn	Sp	Accuracy
VB	89	118	0	3	96.74%	100%	98.57%
MB	86	118	0	6	93.47%	100%	97.14%
SA	80	94	24	12	86.96%	79.66%	82.86%
SE	92	118	0	0	100%	100%	100%
VS	91	116	2	1	98.91%	98.31%	98.57%

Note: TP indicates the true positive, TN indicates the true negative, FP indicates the false positive, and FN indicates the false negative. Sn and Sp indicate sensitivity and specificity, which represent the true positive rate and true negative rate. A proportion threshold of 5% was used for discriminating positive and negative results.

**Table 2:** The BFID markers that could assist the inference of body fluid composition.

Body fluid	BFID markers
MB	MMP10 [41], MMP3 [22]
VB	HBB [42], HBA1 [42], HBA2 [42]
SA	STATH [43], HTN3 [43]
SE	PRM1 [43], PRM2 [43], TGM4 [43], SEMG2 [44]
VS	SPINK5 [45], MUC4 [41]

### Identification of body fluid donors from transcriptome data of mixtures

**Figure 5:**
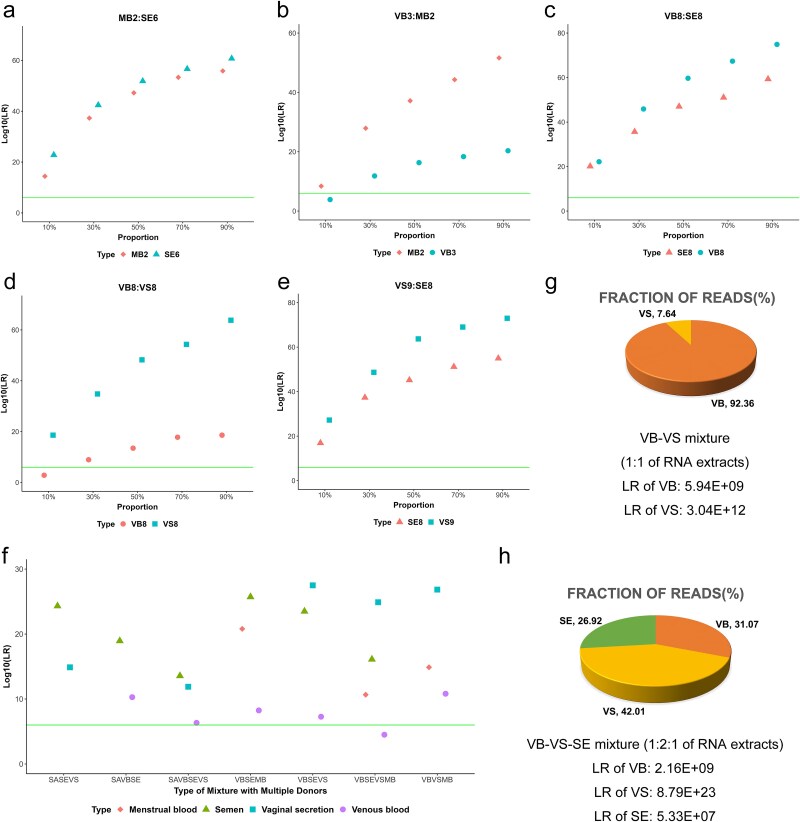
Individual identification of body fluid donors using separated individual SNP profiles from bulk transcriptomes data of multi-body-fluid mixtures involving 2–4 individuals. Individual identification from two-body-fluid (a-e), multi-body-fluid (f) *in-silico* mixtures was statistically illustrated by likelihood ratio (LR) to determine the evidentiary strength of an identity SNP matching for individual identification. Green line represents the 10E+6 LR threshold. (a) Mixtures of menstrual blood and semen. (b) Mixtures of venous blood and menstrual blood. (c) Mixtures of venous blood and semen. (d) Mixtures of venous blood and vaginal secretion. (e) Mixtures of vaginal secretion and semen. (f) Mixtures containing three or four types of body fluids. The deconvolution of body fluid reads fraction and individual identification were also performed on mRNA-seq data of real mixtures (g-h). (g) Mixtures of venous blood and vaginal secretion with a mixed ratio of 1:1. (h) Mixtures of venous blood, vaginal secretion and semen with a mixed ratio of 1:2:1.

With the knowledge of body fluid compositions, the SNPs located in relatively specific genes for a component in the mixture are suitable for genetically identifying this body fluid donor. For two-body-fluid mixtures, only two components existed, and the SNPs within DEGs between these two body fluids could be directly selected as candidate SNPs for individual identification. The top 200 DEGs ordered by decreasing absolute fold changes were applied for SNP selection to ensure that candidate SNPs could be selected with enough specificity compared to another component. We analyzed this SNP selection strategy on five types of two-body-fluid artificial mixtures, including MB-SE, VB-MB, VB-SE, VB-VS and VS-SE mixtures, with read fractions from 0.1 to 0.9 in increasing intervals of 0.2. Saliva-contributed mixtures were not selected for the measurements since saliva transcriptomes were all sequenced from pooled samples and already contained mixed genotypes. The selected SNPs were matched against the reference, namely the original transcriptome data, and LR was applied as a statistical parameter for indicating the strength of evidence. An LR of more than 10E+6 was generally supposed as ‘extremely strong’ evidentiary weight in forensic genetic identification [46]. For most individual matches obtained from two-contributor mixtures, the LR of the matching SNPs was significantly higher than the 10E+6 threshold, greatly favoring the prosecution hypothesis for individual identification (Fig. 5a–e and Supplementary Table S5). However, for minor components representing only 10% of total reads, the number of detected SNPs would be drastically decreased, and some individual matches showed poor performance (Fig. 5b, d). The results suggested the limitation of our deconvolution approach on the imbalanced mixtures.

For multi-body-fluid mixtures, only genes overexpressed in one component compared with any of the other components could be applied for SNP selection. The candidate genes were therefore obviously decreased, and gene selection was extended to the top 250 DEGs to alleviate the impact. The performance of SNP selection and individual matches on these challenging multi-contributor samples was shown in Fig. 5f and Supplementary Table S5. Although the genes for SNP selection decreased, most of body fluid donors from multi-donor mixtures were observed with LRs higher than 10E+6, in which some obtained LRs > 10E+20. Due to the obviously worse performance of individual matches compared with two-body-fluid mixtures, we further decreased the MAF threshold to 0.05 for SNP selection and evaluated the evidentiary weight. The LR values calculated from all contributors exhibited varying degrees of improvement (Supplementary Table S6). These results indicated the abundant information within transcriptome sequencing data, which could be further explored by calibrating the analysis threshold, and demonstrated the capacity of our approach to discriminate a body fluid contributor from others.

### Evaluation of the deconvolution pipeline with mRNA-seq data of real mixture

In contrast to *in-silico* mixtures generated from body fluid transcriptome data, we additionally prepared two real mixture samples and performed mRNA sequencing on these samples. The mixture deconvolution approach was subsequently implemented on these data. As for the VB-VS mixture, only 7.64% of reads were detected from VS, while the remaining 92.36% of reads were all estimated as VB’s reads (Fig. 5g). Although the VB reads showed predominance in this VB-VS mixture, the number of detected SNPs from VB donors was only 41 with a LR of 5.94E+9, compared with the 54 SNPs and a LR of 3.04E+12 from VS donors (Fig. 5g and Supplementary Table S7). No significant difference was shown between two body fluid donors, which might be explained by the phenomenon that VB reads mostly composed of HBB, HBA1 and HBA2 (> 80%). These results indicated the poor RNA quality of this mixture, especially the RNA contributed from VS. On the other hand, the VB-VS-SE mixture obtained 31.07% of reads from VB, 42.01% of reads from VS, and 26.92% of reads from SE (Fig. 5h). This deconvolution result was close to primary proportions of RNA input. The numbers of detected SNPs from the VB, VS and SE donors were 28, 109 and 36, respectively, with LR values ranging from 5.33E+07 to 8.79E+23 (Fig. 5h and Supplementary Table S7). In a word, our results demonstrated the ability of our deconvolution approach to identify body fluid donors from transcriptomes of real mixtures.

### The potential for biogeographic ancestry inference

**Figure 6:**
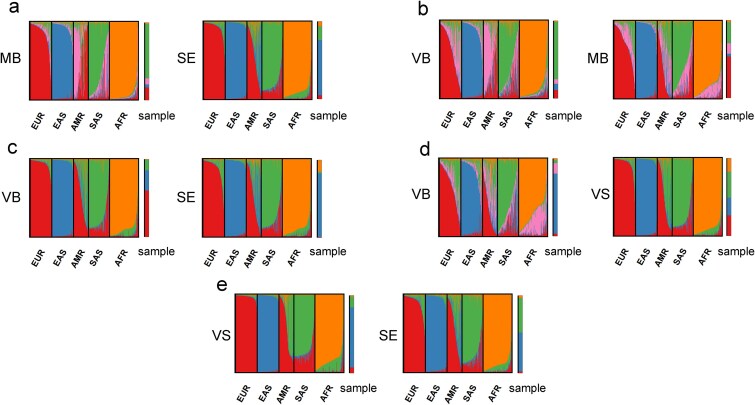
Biparental ancestry analysis using STRUCTURE based on the AISNPs extracted from body fluid donors in two-component balanced mixtures with continental reference populations accessed from 1000 genomes project. (a) The results of MB-SE mixture with inferred donors’ ancestry based on 58 AISNPs for MB and 73 AISNPs for SE. (b) The results of VB-MB mixture with inferred donors’ ancestry based on 24 AISNPs for VB and 55 AISNPs for MB. (c) The results of VB-SE mixture with inferred donors’ ancestry based on 76 AISNPs for VB and 58 AISNPs for SE. (d) The results of VB-VS mixture with inferred donors’ ancestry based on 25 AISNPs for VB and 80 AISNPs for VS. (e) The results of VS-SE mixture with inferred donors’ ancestry based on 95 AISNPs for VS and 49 AISNPs for SE. EUR, Europeans; EAS, east Asians; AMR, Americans; SAS, south Asians; AFR, Africans.

The above results were obtained based on a situation with a known suspect. However, in cases with unknown suspects, the reference profile is unavailable for identity SNP matching, and the identification of mixture contributors through our deconvolution method becomes infeasible. In this situation, the information of biogeographic ancestry may help to narrow down the pool of suspects. We therefore investigated the prospect of our SNP deconvolution strategy to obtain AISNPs for bi-parental ancestry inference. Two-component artificial mixtures with balanced body fluid proportions were applied, and potential AISNPs were selected from relatively specific genes between contributed body fluids. 24 to 95 AISNPs were finally extracted from different body fluid donors. The genetic structure of continental reference populations based on AISNPs extracted from each body fluid donor, as well as their inferred ancestry, was illustrated in Fig. 6. East Asian, European, African, and South Asian populations exhibited their representative ancestral components in the STRUCTURE results from most body fluid donors. Two VB donors were detected with only 24 and 25 AISNPs, and European and South Asian populations also showed a mixed genetic structure in these two results. 5 out of 10 body fluid donors correctly obtained a major East Asian ancestry. The VB donor from VB-SE mixture still obtained a minor East Asian ancestry of 25.81%. The remaining donors all obtained almost no East Asian ancestral components or exhibited a mixed genetic structure.

## Discussion

In this study, we proposed a hypothesis that valuable information present in bulk RNA-seq data would allow for simultaneous determination of the source of body fluid and the identity of contributors in biological mixtures. A novel approach for mixture deconvolution was therefore developed by integrating computational deconvolution methods with SNP-based individual identification, and both *in-silico* mixtures and mRNA-seq data of real mixtures were successfully deconvoluted by the newly proposed method. This proof-of-principle study established the utility of bulk transcriptome data to determine body fluid composition and individually identify the body fluid contributors from a multi-body-fluid mixture. Our methods also showed another advantage: Since the SNPs extracted from transcriptomes are abundant and extensive, they could be easily matched with any forms of datasets, either from genome and transcriptome sequencing or from target sequencing.

Although not physically separating cells of each contributor in mixtures prior to genetic analysis, our approach achieves obtaining individual profiles from bulk RNA-seq data on the basis that SNPs within relatively specific transcripts could directly assign a body fluid component to a specific individual. Compared with traditional methods aiming to interpret mixed DNA profiles, the current method allows for individual identification performed just as single-source analyses, which greatly simplifies the mixture interpretation process. DP > 60 and GQ > 40 were previously demonstrated as appropriate thresholds to obtain highly concordant genotype calls between RNA-SNP and DNA-SNP [24]. In this study, with these filtering thresholds, the number of high-quality SNPs harvested from individual transcriptomes varied a lot. The compositions of microorganisms and RNA degradation are regularly encountered in forensic samples [23, 47]. The former would significantly reduce human-specific reads detected in the data. The latter has been previously reported to significantly influence library complexity and read distribution across exon, intron or intergenic regions in rRNA-depleted libraries [48–50]. In this study, we have observed various human mapping ratios from different samples (Supplementary Table S2). Irregular read distribution or high proportions of duplicate reads were also observed in some samples, indicating the high level of degradation (Supplementary Figs. S1 and S2). All these factors may contribute to a variation in SNP recovery (Detailed in Supplementary Notes). Although some transcriptomes were shown with low quality, most samples obtained enough high-quality SNPs to provide a substantially higher LR than 10E+6, which demonstrated the abundance of high-quality SNPs within transcriptome sequencing data, as well as the feasibility of the data for individual identification [46]. However, for the purpose of mixture deconvolution, SNPs should only be screened from relatively specific RNA molecules, which ensures the accuracy of individual SNP genotypes but also reduces the number of available SNPs. Also, mixtures composed of fewer body fluid types would obtain more SNPs for identifying body fluid donors.

Identifying the origin of contributed body fluids in biological mixtures could yield source-level evidence and provide investigative leads for the contextualization of the contributed stains. The determination of mixture composition was also the first step of our novel approach, and served as a lead to the subsequent SNP deconvolution. In the current study, mathematical methods to deconvolute cell type proportions were introduced for deconvoluting body fluid read proportions, and a quantitative evaluation was performed to explore the impact of factors in the body fluid deconvolution task. It is noteworthy that the choice of data transformation has been maintained with a linear scale, which is known for introducing less bias into the deconvolution results compared with other transformations [39, 51]. Combined impact of deconvolution methods and normalization strategies were then interrogated in this study, and we just discovered that two robust regression methods (FARDEEP and RLR) with original unnormalized data returned more superior performance than other combinations (Fig. 4a). Similar phenomenon could also be observed in Cobos’s study [39]. Normalization strategies are widely used to eliminate technology-derived effects, which were displayed as differences in transcriptome size. However, these methods contribute to an uneven scaling effect on gene expression levels and show negative impacts when distinguishing different types of samples [52]. On the other hand, although unnormalized count matrices could be used for deconvolution of mixed profiles, the outliers from these non-scaling data would also affect the results. The robust regression is known for less susceptibility to outliers, which might be the reason for the superior performance of FARDEEP and RLR on raw count matrices. As for the marker selection, we constructed reference matrices of five body fluids based on the DEGs between each pairwise body fluid type, according to the method mentioned in Newman’s studies [34], and determined the number of markers which presented an optimal operating condition. With the strategy of maximum performance, the body fluid deconvolution task finally obtained a high level of concordance and provided valuable information to determine the number of contributors and their origins.

Due to the detection limit of computational method, we set a fraction threshold of 5% to determine the presence and absence of a specific body fluid. High accuracies were achieved for four body fluid types, except for saliva (Table 1). This phenomenon might be attributed to the high level of microbiome occupation, which contributed to the lowest average genome mapping ratio of the saliva transcriptome in this study [47]. The histological similarity between buccal and vaginal mucosa, as well as their similar expression pattern, may also be one of the reasons [53]. Another unreasonable phenomenon was the 100% accuracy for semen (Table 1). The absence of false-positive results might be attributed to the application of fraction threshold, which effectively filtered out low-level false-positive signals. Similar results were also shown by VB and MB. The fractions of semen were always overestimated by computational methods in semen-contributed mixtures (Fig. 4 and Supplementary Table S4), which may explain the semen with no false-negative results. Similar systematical over- or underestimation for specific cell types was also observed in Newman’s study [34], and this estimation bias still needs to be addressed in future work. We therefore recommended using well-validated body fluid-specific markers exemplified in Table 2 to assist in the determination. By virtue of the high specificity of SA-, SE- and MB-specific markers, the expression of these markers may directly indicate the presence of these three body fluids. As for VB, we noticed that the genes encoding three hemoglobin subunits (HBB, HBA1, and HBA2), which were essential for immature erythroblasts, always held leading roles in expression profiles of VB contributed mixtures [54]. This might be an indicator of venous blood. As for VS, however, we could hardly determine its presence based on VS-specific markers when VS and MB both contributed the mixture. Menstrual blood samples were collected from vagina and always contained varying proportions of vaginal secretion. In this situation, the read fraction estimated from computational methods might be more reliable.

In cases where a given suspect was known to the police, we could speculate the body fluid that the suspect may contribute, and extract corresponding SNPs from the transcriptome of mixture to match against a reference dataset. In this study, whole transcriptome data from 20 individual body fluid samples were applied as reference datasets for both *in-silico* mixtures or real mixtures from mRNA-seq. Also, exome or genome data sequenced from the suspect’s DNA sample could also serve as a reference, and the former has been employed in Kulhankova’s study [12]. However, in cases without a known suspect, the application of our approach is expected to be limited due to the lack of databases including genome or transcriptome data. In these situations, genetic characterization of the mixture contributor may provide an investigative lead. Forensic scientists have already attempted to determine the sex, age, or biogeographic ancestry with RNA or transcriptome data [12, 55]. In this study, we successfully deconvoluted AISNPs for body fluid donors in mixtures. The selection of AISNPs was restricted to relatively specific genes between contributing body fluids, and either the number or the ancestry inference informativeness could not be ensured. Therefore, the performance for ancestry inference was relatively poor. However, our results still demonstrated the possibility of our method to extract ancestry information of mixture contributors, and the inference performance might also be improved with more accurate models in future work. Moreover, it is also imperative to construct reference SNP databases, since the inclusion of profiles from suspects or their relatives would greatly enhance SNP-based identification works.

Forensic stains are always extensively degraded, contaminated, and easily susceptible to environmental effects, such as temperature, humidity and sunlight exposure [56]. The affected RNA quality of forensic samples finally results in the variability of expression profiles, as well as body fluid deconvolution results. In our study, the real VB-VS mixture (1:1) was detected with extremely unbalanced body fluid ratios between VB and VS, which might be attributed to the severe degradation and microbiome occupation in the VS sample. Moreover, hemoglobin genes occupied most of the reads from blood. Finally, both VB and VS donors exhibited poor SNP recovery. These results clearly indicated the weak performance of our method on degraded RNA, or samples affected by other factors, and forensic application of our method is still limited.

The present approach offers notable improvements over the existing methods for mixture deconvolution but still presents several limitations. Conventional STR genotyping exhibits limitations on mixture samples due to the mixed DNA profiles generated by several contributors. Physical separation methods, such as differential lysis [6] or DEPArray™ [7], could obtain individual STR profiles, but they are still limited by recovery losses. Single-cell separation strategies further present challenges to the sensitivity of current technologies. In contrast to these STR-based methods, mixture deconvolution based on bulk RNA-seq avoids the complexity of interpreting mixed DNA profiles and low cell recovery from minor contributors. However, its performance on unbalanced mixtures is still restricted by the sequencing depth, and this SNP-based individualization strategy requires reference availability. Compound markers show advantages to overcome the imbalance between donors [15], but they can hardly analyze a mixture of more than two donors. To the contrary, our RNA-based method could still take effect in multi-donor mixtures when the donors contributed different body fluids, but does not function in a mixture composed of the same body fluid. When compared with other RNA-based genotyping approaches, such as target RNA assays [18, 22], our method benefits from genome-wide SNP availability but still presents lower tolerance to noise and variability. Single-cell RNA sequencing makes full use of the available genome-wide SNPs and provides high evidentiary weight in highly complex mixtures [12]. However, this method currently requires intact cells, and our methods would be more scalable for forensic stains. Besides, our research is a proof-of-concept study performed with a small sample size. Therefore, the generalizability of the reference expression matrix and the transferability of our method are both limited. Future works need to optimize the reference matrix with larger cohorts, including samples under diverse conditions (e.g., fresh or aged, exposure to physicochemical effects or not), and evaluate our method on various RNA-seq platforms. The rise and evolution of molecular or analytical techniques may also mature our deconvolution method for degraded samples or unbalanced mixtures in the future.

To conclude, we have successfully demonstrated the availability of bulk transcriptome data to individually identify body fluid donors from mixture samples. We also described a novel approach, which determines body fluid composition through computational deconvolution methods and separates the SNPs corresponding to each body fluid donor from mixture transcriptome data. Our study constructed a primary reference matrix for body fluid deconvolution, and discovered that robust regression-based methods (e.g. FARDEEP and RLR) combined with unnormalized expression profiles performed better in body fluid deconvolution tasks. Efforts to improve the performance and robustness of our method with larger and more diverse cohorts and to overcome the lack of reference databases are underway. The integration of target RNA assay with bulk RNA-seq also deserves exploration, since it might be an effective strategy to obtain more available SNPs from mixture donors.

**Key Points**

- We successfully demonstrated the feasibility of bulk transcriptomes data for deconvoluting biological mixtures contributed from different body fluids.
- A novel approach was proposed for deconvoluting bulk transcriptomes data of mixtures by integrating a series of bioinformatic methods.
- The method could simultaneously determine body fluid composition and contributors from a multi-body-fluid mixture, providing the direct assignment of a body fluid and a specific individual.

## Supplementary Material

Supplementary_Table_S1_bbaf668

Supplementary_Table_S2_bbaf668

Supplementary_Table_S3_bbaf668

Supplementary_Table_S4_bbaf668

Supplementary_Table_S5_bbaf668

Supplementary_Table_S6_bbaf668

Supplementary_Table_S7_bbaf668

Supplementary_Notes_bbaf668

## Data Availability

The transcriptome sequencing data generated in this study are not publicly available due to privacy and legal restrictions, but are available from the corresponding author upon reasonable request. The scripts used to deconvolute individual SNP profiles from mixtures are available in the GitHub repository at: https://github.com/Chris560665/RNAseq_deconvolution.
