# Supplementary material for: Identification of the body fluid donors from mixture stains using bulk transcriptomes data
Source: Brief Bioinform. 2025 Dec 12;26(6):bbaf668. doi: 10.1093/bib/bbaf668 (PMC12700097; doi:10.1093/bib/bbaf668)
Supplement: Supplementary_Notes_bbaf668 [file supplementary_notes_bbaf668.pdf]

Supplementary Notes

To investigate the potential use of RNA-seq data for human identification, we extracted high-quality SNPs from our individual transcriptome datasets to test the strength of evidence it could provide, with a DP threshold > 60 and GQ threshold > 40 and adjustment for linkage disequilibrium. However, significant variations were observed for the number of detected SNP above the analysis threshold and the calculated random match probability. The possible interpretations might be as follows:

Firstly, we observed that the varied number of detected SNPs generally corresponded with the number of reads mapped to human genome, and samples with more reads and higher mapping ratios would certainly obtain more SNPs with a DP threshold > 60 (Supplementary Table S2). The human genome mapping ratios varied a lot among different body fluids. The decreased mapping ratios might be attributed to the composition of microorganisms. Previous studies have suggested that microbiome RNA occupied various amounts of clean data in all body fluids [1, 2]. Saliva showed the most abundance of microorganisms, followed by vaginal secretion. Menstrual blood microbiota might be transferred from the vagina, and seminal microbiota may be the collective results of various urogenital tract microorganisms [3]. The uncertainty proportions of microbiome RNA may further contribute to differences between individuals.

Apart from microbiome occupation, RNA degradation is also regularly encountered in forensic samples due to sampling methods or transportation conditions. Previous studies have suggested higher intronic distributions in rRNA-depleted libraries from highly degraded or FFPE samples, which might derive from the pre-mRNAs that could not be spliced in time and accumulated in cells under the natural degradation conditions [4, 5]. In this study, except for blood, all body fluid types exhibited samples with high intron distributions (Supplementary Figure S1), which might be an indicator of degradation. The intergenic distributions also increased, indicating that the residue of DNA may also contribute to some reads. The detected reads were assigned to broader regions, and therefore fewer SNPs passed the

analysis threshold. Considering that intron and intergenic reads are regularly characterized as reads from non-coding RNA, either reads from pre-mRNAs or DNA residue would mask the true information. We therefore focused on our expression studies at the mRNA level, and only a few of lncRNAs recorded in the Ensembl databases were included in the subsequent analysis to avoid false prediction of lncRNAs.

Finally, some samples in our studies also showed high proportions of duplicate reads. Except for blood, these reads might primarily be derived from the decreased library complexity of degraded samples [6]. As for the high duplicate reads in blood samples, the high expression of hemoglobin may also decrease the library complexity. The duplicate reads would be flagged by GATK and not counted as the SNP sequencing depth, which also reduced the available SNPs above the analysis threshold. This is a technique problem for low-quality forensic samples.

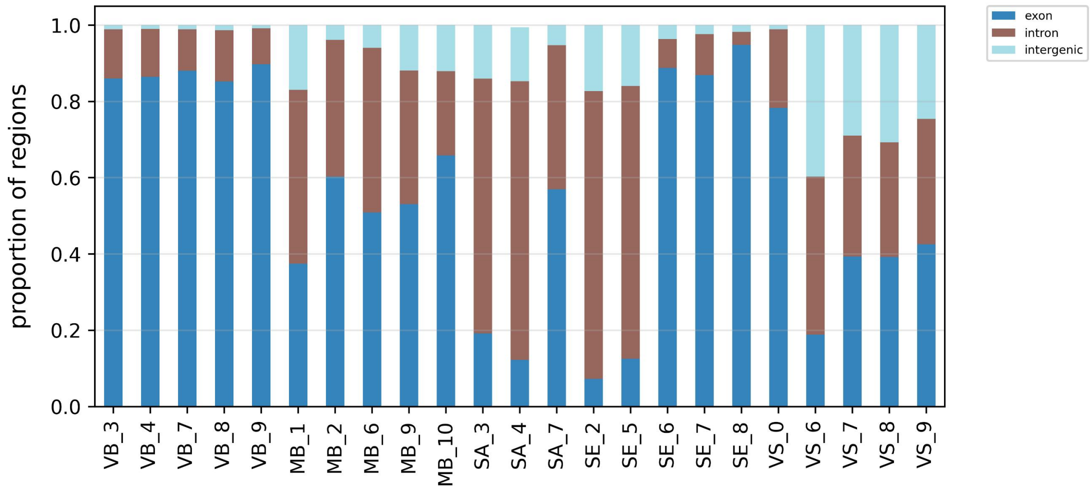

Supplementary Fig. S1 The proportions of exon, intron and intergenic regions in different samples. VB, venous blood; MB, menstrual blood; SA, saliva; SE, semen; VS, vaginal secretion.

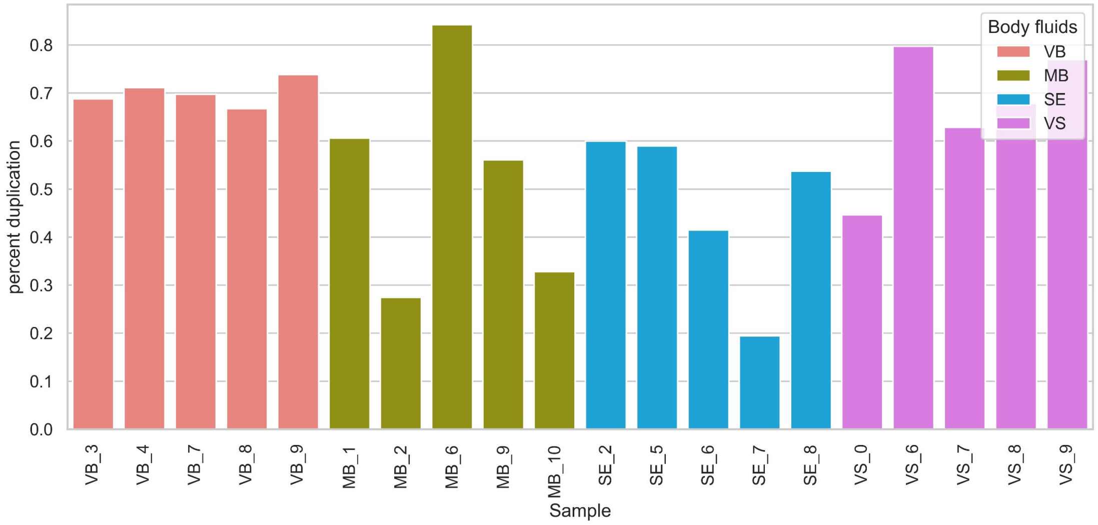

Supplementary Fig. S2 The percentages of duplicate reads marked by GATK in different samples. No result was recorded for saliva, since saliva transcriptomes are not obtained from a single person and are not analyzed by GATK. VB, venous blood; MB, menstrual blood; SE, semen; VS, vaginal secretion.

1. Liu Z, Liu J, Geng J et al. Metatranscriptomic characterization of six types of forensic samples and its potential application to body fluid/tissue identification: A pilot study, Forensic Sci. Int. Genet. 2024;68:102978.
2. Salzmann AP, Russo G, Aluri S et al. Transcription and microbial profiling of body fluids using a massively parallel sequencing approach, Forensic Sci Int Genet 2019;43:102149.
3. Altmäe S, Franasiak JM, Mändar R. The seminal microbiome in health and disease, Nat Rev Urol 2019;16:703-721.
4. Lu W, Zhou Q, Chen Y. Impact of RNA degradation on next-generation sequencing transcriptome data, Genomics 2022;114:110429.
5. Marczyk M, Fu C, Lau R et al. The impact of RNA extraction method on accurate RNA sequencing from formalin-fixed paraffin-embedded tissues, BMC Cancer 2019;19:1189.
6. Shen Y, Li R, Tian F et al. Impact of RNA integrity and blood sample storage conditions on the gene expression analysis, Onco Targets Ther 2018;11:3573-3581.
